# Trichinosis

**Published:** 1890-01

**Authors:** 


### TRICHINOSIS.

The trichinæ, as found in the muscles, are coiled up in small sacs, and while so situated they remain inactive. When taken into the stomach these sacs are acted upon by the gastric fluids, the walls are ruptured and the parasite set free. Passing from the stomach into the intestines, they immediately begin to grow rapidly, and mature in about two days. On the seventh day they give birth to living young, the product of each female parasite being variously estimated to be from two hundred to one thousand. Shortly after birth the young penetrate the mucous membrane, and rapidly make their way to the different muscles throughout the body, which they usually reach in about fourteen days. Once in the muscles, they soon become inclosed in sacs or capsules which are formed around them. They are then inactive, but retain their vitality for years, sometimes even until after the death of the person infected. After a time there are deposited in these sacs certain lime salts, more especially the carbonate of lime. The fluid within becoming milky, the capsules are then visible to the naked eye of even inexperienced observers as small white dots or streaks. According to some authorities, under certain circumstances. not yet positively determined, the trichinæ may die and decay, or a deposition of lime salts may cause them to petrify and break into pieces ; then their former nature can be recognized only by the peculiar position occupied by the fragments.

When the flesh of a trichinous animal containing the living parasite is eaten by man it produces the disease called trichinosis. The earliest symptoms are referable to the digestive system, and are chiefly due to the perforations of the mucous membrane by the young trichinæ. "It is not difficult," says Flint, "to understand that the aggregated punctures of the mucous membrane by these parasites should occasion notable disturbance. when it is considered that the trichinæ which have been found to be contained in a half pound of meat may be sufficient to give birth in a few days to a brood numbering thirty million."

The first symptoms of trichinosis generally occur within ten days following the eating of meat containing trichinæ ; that is, as soon as the young are developed and start on the journey to the muscles. Loss of appetite, nausea, vomiting, abdominal pains, and diarrhœa are characteristic of the disease, when present in a high degree. With these are associated a feeling of general weakness, slight fever, and occasionally dizziness. Some patients complain of an unpleasant pasty taste ; not infrequently the breath is extremely offensive. After a short time there are wandering pains in the limbs and a general muscular lameness : the muscles then become stiff and painful to the touch, and are hard and more or less swollen. The pain is but trifling while rest is enforced ; but movements of the body and limbs cause great suffering. The difficulties of chewing and swallowing are often very great, and not infrequently only liquid nourishment can be taken. Owing to the pain, which, in very severe cases, movement causes, the patients shrink from changing their position, and lie with their knees drawn up and arms sharply bent, presenting a picture of the greatest helplessness. Puffiness of the eyelids is apt to occur ; in some cases the whole face becomes swollen, and the lower extremities dropsical.

A marked characteristic of trichinosis is profuse sweating ; loss of voice and deafness occasionally result. The nervous system shares in the general disturbances, and sleeplessness is a very prominent symptom. Delirium, while not constant even in the severer cases, is present in some ; more often the mental derangement is manifested by a condition of apathy, a marked indifference of the patient to his surroundings. When recovery takes place, all the symptoms gradually abate, and generally disappear in from four to five weeks, although they may linger for a much longer period. When death occurs it is usually during the fourth week, and may be due to exhaustion, or, as is quite often the case, to insufficiency of respiration, a consequence of the invasion of the respiratory muscles by a large nnmber of trichinæ.

The hog is supposed to be the peculiar and original bearer of trichinæ; in it the whole course of evolution of the trichinæ takes place; in it the trichinæ are propagated from generation to generation, and from it, as a rule, man, the rat, and the cat derive their trichinæ. It is generally assumed that he may require trichinæ in different ways peculiar to his habit of feeding on the waste of slaughter-houses and gutters. It is believed that "the flaying yards, in which swine are crowded, are prolific breeding places for trichinous hogs."

The process of smoking, pickling, or salting cannot be relied upon for the destruction of this worm, therefore, only by submitting pork to a sufficiently high temperature can protection against infection be secured. It is generally accepted that trichinæ are surely killed by a temperature of one hundred and fifty-five degrees Fahr. In cooking masses of meat this degree of heat is obtained in the interior, especially in the neighborhood of bones, only after long roasting or boiling.—*Ext.*

